# Kidney Function Assessment in African American Patients: A Narrative Review for Pharmacists

**DOI:** 10.3390/pharmacy10030065

**Published:** 2022-06-20

**Authors:** Dhakrit Rungkitwattanakul, Weerachai Chaijamorn, Eunice Han, Mohammed Aldhaeefi

**Affiliations:** 1 Department of Clinical and Administrative Pharmacy Sciences, Howard University College of Pharmacy, Washington, DC 20059, USA; mohammed.aldhaeefi@howard.edu; 2 Faculty of Pharmacy, Siam University, Bangkok 10160, Thailand; weerachai.cha@siam.edu; 3 United Healthcare, Minnetonka, MN 55305, USA; eunicehan85@gmail.com

**Keywords:** glomerular filtration rate, kidney function, race, African American, black, equations

## Abstract

Recent recognitions of longstanding societal inequity in kidney function assessments have prompted the call to eliminate race as part of the algorithm to assess estimated glomerular filtration rate (eGFR). Previous equations for eGFR estimation adopted race as part of the calculation. Incorporating race within eGFR equations results in overestimating and underestimating Black and nonblack patients, respectively. The inclusion of race is controversial. In September 2021, the National Kidney Foundation (NKF) and the American Society of Nephrology (ASN) combined task force recommended estimating the kidney function without using a race variable. The task force endorsed race-free creatinine-cystatin C equations to be more accurate than the creatinine-only equations. Before the application of NKF-ASN revised recommendations, major healthcare disparities influenced daily clinical practice. Those disparities include the delay in initiating medications that have reanl or cardio-protective effects, such as sodium-glucose cotransporter–2 inhibitors (SGLT-2i) and angiotensin-converting enzyme inhibitors (ACEIs). Clinical judgment should be employed when dose adjusting medications. Combining the eGFR with other clinical assessment tools such as urinary output, the expanded use of confirmatory tests, and the eGFR trend is suggested for a better kidney function assessment. Additionally, creatinine-cystatin C is recommended when feasible, and when institutions have the laboratory abilities.

## 1. Introduction

Chronic kidney disease (CKD) is one of the most common medical conditions affecting all age groups and is a worldwide public health problem. In the United States (US), reports from the United States Renal Data System (USRDS) in 2019 showed the number of patients enrolled in the End-Stage Renal Disease (ESRD) Medicare-funded program increased from 17,907 in 1980 to 746,557 in 2017. The increase was almost a 3% increase from the previous year [1]. The report also calls attention to the striking racial variations in the prevalence of CKD and ESRD. In 2017, the incidence rate for ESRD in the United States was 370.2 per million/year. Of those, the rate among African-American patients was about three times greater than the rate among the Caucasian population [1]. Similarly, patients of African descent suffered a higher incidence of ESRD in the United Kingdom (UK) [2]. There is also a significant racial disparity in the etiologies of ESRD, average time of pre-ESRD nephrology care, and proportion of fistula use. Furthermore, the age of patients with ESRD tends to be younger among African-American patients [1]. This has drawn attention to the consequences of health disparities and inequity in medicine among the medical community.

African-American or Black patients are at a higher risk of developing CKD. The incidence of hypertension, diabetes, and obesity, which are the traditional risk factors for CKD is much higher in the African-American population than other races [3]. Additionally, among ethnic minority communities, lack of access to care and social determinants of health are the most likely explanations of unfavorable outcomes [4]. Accuracy in assessing glomerular filtration rates (GFRs) is central to clinical practice and research. In clinical practice, GFR is used to assess and interpret clinical signs and symptoms of kidney diseases. The accurate assessment of GFR is also important for drug dosage adjustments and to detect, stage, and manage both acute kidney injury (AKI) and CKD. In research, GFR is used to stratify patients, or can be used as an outcome. In clinical practice, the diagnosis of AKI or CKD are independent of race. Conversely, estimating GFR (eGFR) based on serum creatinine (eGFR_cr_) requires the identification of race and is recommended and endorsed by the current practice guidelines [5]. In 2019, Eneanya et al. suggested using race in eGFR calculations in cases where it confers substantial benefits, i.e., improving accuracy. Currently, there are limited data to demonstrate the outcome benefits of using race as part of the calculation [6]. Using race as a variable may cause problems for transparency and accuracy. In the US, about 3% of individuals identified themselves in the multiracial category in the 2020 census [7]. Individuals with mixed race would find it challenging to identify their race and it would affect the estimation of GFR. A key example for this discrepancy is the access to nephrological care. In the US, patients with GFR of less than 30 mL/min/1.73 m^2^ are referred for nephrology care. Take, for instance, a hypothetical 45-year-old male patient with a serum creatinine of 2.5 mg/dL, whose father self-identifies as White and mother self-identifies as Black. If this patient was considered to be Black, based on the 2009 CKD-EPI equation, his eGFR would be 36 mL/min/1.73 m^2^. If he was considered to be White, his eGFR would be 30 mL/min/1.73 m^2^, which would lead to a nephrology referral. Based on USRDS report in 2019, African-American patients were provided with significantly less time during pre-ESRD care by nephrologists [1]. These scenarios show the importance of the accuracy of estimating kidney function and the need to reconsider the use of race in calculating eGFR. In this article, we aimed to review the limitations of existing measures to estimate kidney function and discuss the recent development in assessing the eGFR.

## 2. Evolution of Equations to Estimate Kidney Function

It is widely agreed that assessing kidney function or GFR is a prerequisite in all clinical practice. Unfortunately, an accurate GFR cannot be measured directly in humans; instead, it is measured by the clearance of markers or substances eliminated from plasma and is filtered through the glomerulus. Exogenous markers such as inulin, iohexol, or iothalamate have been used to measure GFR [8]. However, it is costly and not readily available to most clinical practices. To promote its feasibility and applicability, the clearance measurements of endogenous markers such as creatinine were suggested. In 1976, Donald W. Cockcroft and M. Henry Gault developed the first formula to predict creatinine clearance from serum creatinine by comparing it with urinary clearance. The equation was derived from observations made by examining data from 249 male patients whose ages ranged from 18–92 years, and were primarily Caucasians in Canada [9]. The equation requires age, gender, weight, and serum creatinine to estimate creatinine clearance (eCrCl) in milliliters per minute (mL/min). Before the year 2000, attempts to study approaches to slow the progression of chronic kidney disease were carried out. At the time, a low protein diet was found to be beneficial, hence the study “Modification of Diet in Renal Disease, MDRD”. The investigator collected patients’ data to determine the outcomes of the modified diet and was able to create a formula to estimate GFR based on the availability of data. The accuracy of the equation was also compared with the measured GFR (mGFR) from the study participants, ensuring the accuracy of the new formula. Of the 1628 patients enrolled in the MDRD study, 88% were White [10]. The equation requires age, gender, serum creatinine, and race to estimate eGFR in mL/min/1.73 m^2^. They also found that at any given GFR, the serum creatinine level is significantly higher in Black than White populations. Therefore, to improve the accuracy of the equation, the multiplication factor of 1.18 was added to the formula [10]. This is the first time that race was introduced as part of the eGFR estimation. However, the study was primarily conducted in CKD patients, thus affecting the accuracy when used in patients with normal or near normal kidney function.

**Figure 1:**
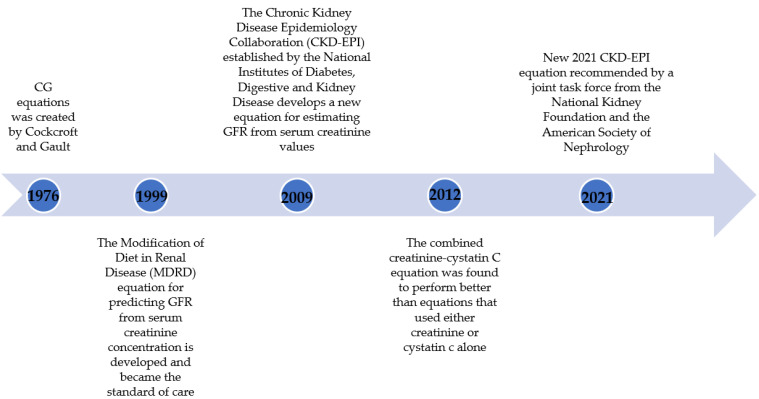
Historical perspective of kidney function assessment.

In 2009, the Chronic Kidney Disease Epidemiology Collaboration (CKD-EPI) was established by the National Institutes of Diabetes Digestive and Kidney Disease. The group developed a new equation suitable for all patient populations (severely reduced, reduced, and normal GFR). The new equation promises more accuracy than the MDRD study equation. One key limitation was noted in that there was limited representation of all racial and ethnic minorities across the study population of 8254 patients [11]. The CKD-EPI equation requires age, gender, serum creatinine, and race to estimate eGFR in mL/min/1.73 m^2^, similar to the MDRD equation. Comparably, the multiplication factor of 1.159 was still utilized [11]. In 2012, Inker et al. suggested the inclusion of serum cystatin C to the CKD-EPI equation to increase the performance, and it was found to be better than equations that used either creatinine or cystatin C alone [12]. Cystatin C is cysteine protease inhibitor that is commonly found in all nucleated cells and is produced at a constant rate, filtered freely, and not secreted into the renal tubules or reabsorbed back to the blood stream [13,14]. However, cystatin C is still affected by several factors. Body weight, age, gender, thyroid function, and glucocorticoid use are the known factors affecting serum cystatin C level, albeit the effect is less than observed with serum creatinine [13,14]. Due to the attractive characteristic of cystatin C, its use was encouraged in clinical practice. However due to the higher cost and availability of the assay, the use of cystatin C in clinical practice is not universally implemented (Figure 1) [9,10,11,12], Table 1 [9,10,11,12].

Of note, there are other equations to estimate eGFR (Salazar-Corcoran, Jelliffe, and Full Age Spectrum (FAS)) [15,16,17]. They are not mentioned in this review due to not being used extensively in clinical practice.

## 3. Race Coefficients in Equations Estimating GFR

Generally, the serum creatinine concentration is affected by renal and non-renal pathways. The concentration is inversely related to the degree of renal function or GFR and directly related to non-renal pathways that affect the level, called “non-GFR determinants”, which include liver function, generation of creatinine, and renal tubular function. It is difficult to quantify non-GFR determinants since they are related to demographic factors such as weight, height, gender, race, diet, or medication [18]. To improve GFR estimation methods, it is important to identify particular subgroups of populations that possess variations of serum biomarkers. The 2009 CKD-EPI creatinine equation includes age, gender, and race (identified as Black versus nonblack) as variables in addition to serum creatinine. Based on the dataset, Black individuals had 15.9% higher serum creatinine compared with White individuals at a comparable mGFR (Table 2) [10,11,12]. The observation prompted investigators to suggest that the higher serum creatinine could be explained by a higher muscle mass among Black individuals, rather than a reduced kidney function. Thus, calculating eGFR among Black individuals requires the correction coefficient of 1.59, which leads to higher GFR values in Black individuals as a result. The addition of race is controversial for several reasons. First, there is genetically one human species “*Homo sapiens*”. Multiple analyses showed that all human populations are genetically similar and belong to “*Homo sapiens*” [19]. Race was never classified as a subspecies. Therefore, using race to differentiate individuals has no biological basis. Second, this relies on the assumption of Black patients in MDRD and CKD-EPI studies as African American. The designation of “African American” does not exist. This proposition disregards all substantial variability among Black individuals. In one study, Flamant et al. examined the performance of GFR-estimating equations among African Europeans. The black coefficient in CKD-EPI (15.9%) was excessive for Black Europeans, suggesting only an 8% higher in serum creatinine compared to Caucasian individuals with similar mGFR [20]. Additionally, using the Black race coefficient does not perform well in Africa. A study using a dataset from the Congo and Ivory Coast showed CKD-EPI creatinine performed poorly. The percentage of eGFR = within 30% of mGFR, P_30_, was only 64% [21]. Another dataset based on a healthy Congolese population also demonstrated both MDRD and CKD-EPI equations using creatinine with the race coefficient overestimated mGFR by 17.9 ± 19.2 and 14.5 ± 27.1 mL/min/1.73 m^2^, respectively. However, the bias was improved with the use of cystatin C [22]. Overall, a race coefficient was either small or not required in the CKD-EPI equation using either both creatinine and cystatin C or cystatin C alone, thereby questioning the generalizability outside the United States. Third, the ethnicity of study populations in the MDRD study was reportedly assigned by study personnel, without explicit criteria, and probably examined by skin color of the participants [23]. Nonetheless, the statement was later modified by the investigators to “ethnicity was self-identified by the participants” in 2021 [23,24]. The inclusion of race in eGFR estimations may have been a result of the intention to achieve statistical precision in the calculation, rather than accounting for the social and biological constructs of the populations [25]. These discrepancies and limitations should not be ignored and underlines the flaws of using race as part of estimating GFR.

## 4. Markers in eGFR Calculations and Consequences of Removing Race from GFR Estimation

**Figure 2:**
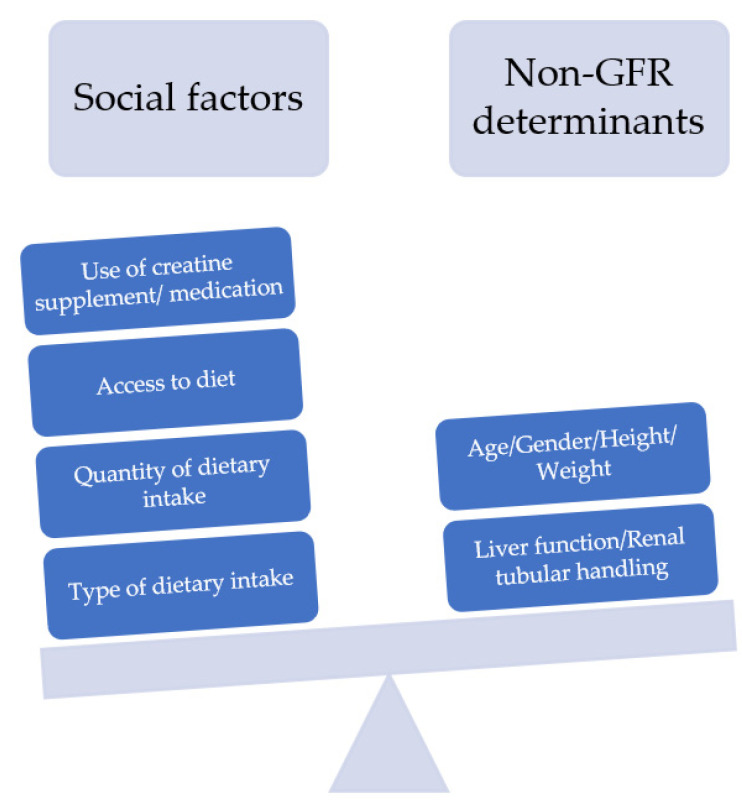
Factors affecting serum creatinine level, showing levels of serum creatinine can be influenced by several social factors which are highly variable among all populations, including all racial background.

Creatinine and cystatin C are the most widely used biomarkers in clinical practice for eGFR calculation. The limitations of using serum creatinine as a filtration marker has been examined extensively, namely in terms of the variation in creatinine production and secretion [26]. Several non-GFR determinants and social factors have been implicated to affect the level of serum creatinine (Figure 2) [13,19].

Efforts have been made to create an eGFR estimating equation without using race. Novel filtration markers such as cystatin C or beta-2 microglobulin have been considered in place of race. Inker et al. reported that serum cystatin C is less affected by race [12]. Remarkably, using both serum creatinine and cystatin C improves the accuracy of the eGFR calculation while reducing the impact of the race coefficient from 15.9% to 8% [12]. Despite this, the capacity to attain accuracy in the GFR estimation without applying the race coefficient from most available filtration markers alone is still limited as they are not able to fully capture the distinctions related to non-GFR determinants. Several attempts have been made to find additional markers to improve the accuracy of estimating GFR when race is removed from the calculation. In the CRIC study, adding body surface area or the incorporation of genetic ancestry data instead of race did not improve the accuracy in the serum creatinine-based eGFR calculation [27]. They also found that the precision and validity of the estimation of GFR from serum cystatin C were comparable to the estimation based on the serum creatinine without the requirement of race or genetic ancestry data. Further, the accuracy of eGFR calculated based on cystatin C was not increased nor decreased by inclusion of race [27]. The results are consistent with previous studies [12,28]. In 2020, Levey et al. evaluated the performance of the 2009 CKD-EPI equation in estimating mGFR by eliminating race and substituting it with height and weight. The performance of the equation was evaluated using root mean square error (RMSE) and bias (Table 3). Similarly, utilizing height and weight instead of race did not improve the accuracy (RMSE increases from 0.236 to 0.43) in both Black and non-Black individuals (Table 3) [25]. This prompted the search for the new equation to better estimate eGFR without race.

## 5. Reassessment of the Use of Race in eGFR Estimating Equation and the New 2021 CKD-EPI Equation

In July 2020, the National Kidney Foundation and the American Society of Nephrology (NKF-ASN) formed a joint task force to reassess the inclusion of race for eGFR estimates [29]. The task force was a multidisciplinary and diverse team including experts such as nephrologists, pharmacists, medical geneticists, biostatisticians, and laboratory medicine specialists. The task force worked over 10 months to evaluate approaches to address the use of race in GFR calculation and issue recommendations. The task force comprehensively evaluated each approach considering the following six attributes: assay availability and standardization; implementation; population diversity in equation development; performance compared with measured GFR; consequences to clinical care, population tracking, and research; and patient centeredness. The task force recommended the immediate implementation of the new 2021 CKD-EPI creatinine equation refit without the race variable in all laboratories in the United States [29]. The recommendation was primarily based on the study led by Inker et al. [30]. They developed the eGFR estimation without race and validated this in a dataset. In the validation study, P_30_ (the proportion of eGFR within 30% of mGFR) and the correct classification (the agreement between mGFR and eGFR categories of CKD) were used to evaluate the accuracy of the equation. In the 2021 equation using serum creatinine, age, and sex, the accuracy of estimating eGFR compared with mGFR improved (P_30_ 85% to P_30_ 87%). Further, new creatinine-cystatin C equations without race were more accurate than the creatinine only equation (Table 4) [30]. The differences in variables and coefficients in the equations are shown in Table 5 [11,30].

## 6. Implications of the Change on Pharmacy Practice

Estimating GFR is not only beneficial for diagnostic and treatment purposes. The consideration of eGFR or kidney function is required for medication initiation, adjustment, or discontinuation. Historically, the United States Food and Drug Administration (FDA)-mandated pharmaceutical industry to provide renal dosing for medications that are primarily excreted renally including antibiotics, antidiabetics, and chemotherapeutic agents based on the CG equation [31]. Over the past decade, several studies found the limitations of estimated creatinine clearance (eCrCl) based on CG equation i.e., discordance of eCrCl with mGFR, issues with a standardized assay of creatinine, and the demographics population studied [32,33,34]. The Kidney Disease: Improving Global Outcomes (KDIGO) in 2010 suggested using the most accurate method to assess the kidney function for the individual patient including eGFR or mGFR for drug dosing, not limited to just eCrCl from the CG equation [35]. More recently, the FDA addressed this issue in their guidance for the pharmaceutical industry in 2020, suggesting the use of eGFR assessment for renally excreted drugs moving forward [36]. They also suggested using “any contemporary, widely accepted, and clinically applicable estimating equation for the population being studied is considered reasonable to assess kidney function” [36]. It is also important to note that drug clearance is proportionate to individual eGFR, which is expressed as mL/min, and not body surface area (BSA) indexed eGFR in mL/min/1.73 m^2^. Therefore, the eGFR should be adjusted to individualize for drug dosing in patients with a BSA different to the standard (1.73 m^2^).

Previously to the newly published NKF-ASN task force recommendations, major disparities in practice were found when treating minorities, such as Black patients, due to the eGFR overestimation from the race coefficient [29,37]. These disparities include the delay in the initiation of kidney-protective medications such as sodium-glucose cotransporter–2 inhibitors (SGLT-2i), angiotensin converting enzyme inhibitors (ACEIs), or angiotensin receptor blockers (ARBs), less likely to be diagnosed with CKD, less likely to be eligible for kidney transplantation, or receiving insurance coverage for medication and kidney disease education [38]. With the new 2021 equation, a higher number of Black patients will benefit from the early initiation of kidney protective medications. However, inappropriate dose reduction and medication discontinuation would also increase when treating Black patients due to a decrease in the eGFR value. Patients who experience a drop in their eGFR resulting in reclassifying them to a different dosing recommendation threshold should receive a careful consideration of risk versus benefit ratio of the medication. For metformin, the revised FDA’s dosing recommendation was to avoid use in eGFR < 30 mL/min/1.73 m^2^ and suggested caution for eGFR 30–45 mL/min/1.73 m^2^. The decision to discontinue metformin must be individualized by assessing the risk of lactic acidosis based on predisposing conditions. Nephrotoxic medications should be dosed carefully in relation to the risk for accumulation and the side effects of supratherapeutic levels. Whenever possible, therapeutic drug monitoring should be utilized. Vondracek et al. suggested careful considerations be placed on patients at a high risk for developing side effects, receiving high-risk medications or receiving medications for high-risk conditions, and that the results from various methods of eGFR calculation should be considered so the estimate that can best balance the risks and benefits from dosing adjustments is selected [39].

Overall, clinicians should use their clinical judgment and experience with a thoughtful risk–benefit calculation when initiating or dose-adjusting medications among patients with CKD, especially with a proven long-lasting renal or cardio-protective effect. Clinicians should continue the practice of not relying on eGFR alone to continue or discontinue the medication; rather, the eGFR should be considered in combination with a clinical assessment of symptoms and the expanded use of confirmatory tests. The use of eGFR for drug dosing should not be limited to one single estimate, but an evaluation of the trend along with other assessments should be performed. Utilizing both creatinine and cystatin C to estimate eGFR is recommended before adjusting medication when feasible. Additionally, establishing proper follow-up and monitoring for kidney function parameters is helpful when considering medication initiation and dose adjustments.

## 7. Conclusions

Every component of our society is taking a careful consideration of race and racism. Pharmacy and Medicine are no exception. Just as in other aspects of healthcare, there are a number of examples of health disparities affecting clinical practice. Utilizing race to quantify kidney function has no biological ground and is an imperfect surrogate parameter as race is a social construct, not a biological construct. The NKF-ASN task force suggested removing race as part of the eGFR equation and published a new race-free eGFR equation. The new 2021 equation that utilizes serum creatinine and cystatin C without race is more accurate than each marker alone and led to smaller differences between non-Black and Black individuals. The immediate implementation of the new 2021 equation is encouraged.

## Figures and Tables

**Table 1:** Equations to estimate GFR.

Equation	Year	Formula	Parameter
Cockcroft and Gault	1976	((140 − age) × weight)/(72 × Scr)	Age, sex, weight, serum creatinine
Modification of Diet in Renal Disease (MDRD)	1999	GFR = 175 × Serum Cr^−1.154^ × age^−0.203^ × 1.212 (if patient is black) × 0.742 (if female)	Age, sex, race, serum creatinine
Chronic Kidney Disease Epidemiology (CKD-EPI)-creatinine	2009	A × (Scr/B)^C^ × 0.993^age^ × (1.159 if black), where A, B, and C are the following: Female: if Scr ≤ 0.7: A = 144, B = 0.7 C = −0.329. if Scr > 0.7: A = 144, B = 0.7, C = −1.209. Male: if Scr ≤ 0.9: A = 141, B = 0.9 C = −0.411. if Scr > 0.9: A = 141, B = 0.9, C = −1.209	Age, sex, race, serum creatinine
Chronic Kidney Disease Epidemiology (CKD-EPI)-creatinine-cystatin C	2012	133 × (Scys/0.8)^A^ × 0.996^age^ × B, where A and B are the following: Female: if Scr ≤ 0.8: A = −0.499, B = 0.932 if Scr > 0.8: A = −0.499, B = 0.932. Male: if Scr ≤ 0.8: A = −0.499, B = 1.0 if Scr > 0.8: A = −0.499, B = 1.0	Age, sex, race, serum creatinine, serum cystatin C

**Table 2:** Coefficients in MDRD and CKD-EPI equations.

Equation	Marker	Year	Age	Gender-Women	Black Race
MDRD	Creatinine (eGFRcr)	1999	Age^−0.203^	0.74	1.21
CKD-EPI	Creatinine (eGFRcr)	2009	0.993^Age^	0.75	1.159
CKD-EPI	Cystatin C (eGFRcys)	2012	0.996^Age^	0.93	NA
CKD-EPI	Creatinine-cystatin C (eGFRcr-sys)	2012	0.995^Age^	0.83	1.08

**Table 3:** Performance of using body size in predicting eGFR in the 2009 CKD-EPI equation.

	All Individuals (*N* = 8254) ^a^		Black Individuals (*n* =2601)
Coefficients used in the equation ^a^	Black race coefficient	Root mean square error	Root mean square error
		(95% CI) ^b^	(95% CI)
Serum creatinine, age, sex, race	1.16	0.236	0.243
		(0.229 to 0.242)	(0.232 to 0.254)
Serum creatinine, age, sex	N/A	0.244	0.258
		(0.238 to 0.251)	(0.248 to 0.268)
Serum creatinine, age, race, sex, height, and weight	1.15	0.235	0.242
		(0.229 to 0.242)	(0.232 to 0.253)
Serum creatinine, age, sex, height, and weight	N/A	0.243	0.255
		(0.237 to 0.250)	(0.245 to 0.265)

^a^ Data are from the CKD-EPI pooled development datasets; ^b^ RMSE is the square root of the mean of squared differences between mGFR and eGFR; lower RMSE values indicate higher accuracy of the eGFR. 95% CI, 95% confidence interval.

**Table 4:** Comparison of eGFR and mGFR agreement based on 2021 CKD-EPI equation.

Markers and Non-GFR Determinants Used	P_30_	P_30_ % Difference between Black and Non-Black	Correct Classification
2009 CKD-EPI-Scr, Age, Sex, Race	Black 85%	−4%	Black 63%
	Non-Black 89%		Non-Black 69%
**2021** CKD-EPI-Scr, Age, Sex	Black 87%	1%	Black 62%
	Non-Black 86%		Non-Black 67%
2009 CKD-EPI-Cys-C Age, Sex	Black 89%	−3%	Black 68%
	Non-Black 92%		Non-Black 71%
**2021** CKD-EPI-Cys-C/Scr Age, Sex	Black 90.5%	−0.3%	Black 68%
	Non-Black 90.8%		Non-Black 70%

P_30_ is the proportion of eGFR within 30% of measured GFR. Correct classification refers to agreement between measured GFR and eGFR categories of more than 90, 60 to 89, 45 to 59, 30 to 44, 15 to 29, and less than 15 mL per minute per 1.73 m^2^.

**Table 5:** Differences in variables and coefficients in the 2009 and 2021 CKD-EPI equations.

Equation *	Intercept μ (95% CI)	Coefficients for Creatinine (95% CI) **		Coefficient c for Age (95% CI)	Coefficient d for Female Sex (95% CI)	Coefficient e for Black Race (95% CI)
		*a*_1_	*a*_2_			
2009 CKD-EPI creatinine	141	F: −0.329 (−0.428 to −0.230); M: −0.411 (−0.508 to −0.314)	−1.209	0.9929	1.018	1.159
	(139 to 144)		(−1.220 to −1.198)	(0.9925 to 0.9933)	(1.007 to 1.029)	(1.144 to 1.170)
2021 CKD-EPI creatinine (without race)	142	F: −0.241 (−0.344 to −0.138); M: −0.302 (−0.403 to −0.202)	−1.200	0.9938	1.012	---
	(139 to 144)		(−1.211 to −1.189)	(0.9935 to 0.9942)	(1.000 to 1.023)	

* The equations are referred to by the filtration marker (creatinine) and the demographic factors (age, sex, and race or age and sex) that were used in their development. ** The coefficient *a*_1_ is used for levels of creatinine less than or equal to 0.9 mg per deciliter for male participants and 0.7 mg per deciliter for female participants. The coefficient *a*_2_ is used for levels of creatinine greater than 0.9 mg per deciliter for male participants and 0.7 mg per deciliter for female participants.

## Data Availability

Not applicable.
